# Changes in Antioxidant Defense System Using Different Lipid Emulsions in Parenteral Nutrition in Children after Hematopoietic Stem Cell Transplantation

**DOI:** 10.3390/nu7095335

**Published:** 2015-08-28

**Authors:** María Auxiliadora Baena-Gómez, María José De La Torre Aguilar, María Dolores Mesa, Juan Luis Pérez Navero, Mercedes Gil-Campos

**Affiliations:** 1 Department of Paediatrics, Reina Sofia University Hospital, Institute Maimónides of Biomedicine Investigation of Córdoba (IMIBIC), University of Córdoba, Avda Menéndez Pidal s/n, 14004 Córdoba, Spain; E-Mails: mabaenagomez@gmail.com (M.A.B.-G.); delatorremj@orange.es (M.J.T.A.); juanpereznavero@hotmail.com (J.L.P.N.); 2 Department of Biochemistry and Molecular Biology II, Institute of Nutrition and Food Technology, Centre of Biomedical Research, University of Granada, Avda del Conocimiento, 18071 Granada, Spain; E-Mail: mdmesa@ugr.es

**Keywords:** hematopoietic stem cell transplantation, parenteral nutrition, lipid emulsions, fish oil, soybean oil, fatty acids, antioxidant status, oxidative stress, alpha-tocoferol, children

## Abstract

Background: Traditionally, lipids used in parenteral nutrition (PN) are based on ω-6 fatty acid-rich vegetable oils, such as soybean oil, with potential adverse effects involving oxidative stress. Methods: We evaluated the antioxidant defense system in children, after hematopoietic stem cell transplantation (HSCT), who were randomized to use a lipid emulsion with fish oil or soybean oil. Blood samples at baseline, at 10 days, and at the end of the PN were taken to analyze plasma retinol, α-tocopherol, β-carotene, coenzyme Q9 and coenzyme Q10 levels, and catalase (CAT), glutathione reductase (GR), glutathione peroxidase (GPOX), and superoxide dismutase (SOD) levels in lysed erythrocytes. Results: An increase in plasma α-tocopherol levels in the group of patients receiving the fish oil-containing emulsion (FO) compared with the group receiving the soybean emulsion was observed at day 10 of PN. Concurrently, plasma α-tocopherol increased in the FO group and β-carotene decreased in both groups at day 10 compared with baseline levels, being more significant in the group receiving the FO emulsion. Conclusion: FO-containing emulsions in PN could improve the antioxidant profile by increasing levels of α-tocopherol in children after HSCT who are at higher risk of suffering oxidative stress and metabolic disorders.

## 1. Introduction

Hematopoietic stem cell transplantation (HSCT) entails the infusion of marrow cells preceded by the administration of toxic chemotherapy (therapy). The conditioning regimen not only decreases the tumor burden but also maximizes the capability of the donor cells to engraft successfully by suppressing the patient’s immune system [1]. This aggressive chemotherapy affects the digestive system and limits oral food intake. Parenteral nutrition (PN) is the main feeding method when oral tolerance is poor. However, the clinical benefits of PN in patients receiving high-dose chemotherapy during HSCT are unknown [2,3].

The role of oxidative stress (OS) is well known in the pathogenesis of acquired malnutrition [4,5]. Conditioning therapy given to HSCT recipients increases the production of reactive oxygen species and decreases the concentration of antioxidants and certain enzymes, vitamins E and C, and β-carotene [2,6,7]. This is probably responsible for the acute and delayed toxic effects that cytostatic drugs may have on tissues such as the gastrointestinal system, lungs, liver, or bladder [7]. Regardless of the conditioning therapy, HSCT itself can disturb the pro-oxidant/antioxidant balance [6].

Therefore, it appears that a significant subgroup of PN patients needs more antioxidant molecules to avoid the increased oxidant status [2]. To evaluate these changes, the quantification of antioxidant capacity is possible to reflect the antioxidants’ vulnerability to an oxidation state. The body’s antioxidant defense mechanism includes enzymatic and non-enzymatic molecules as well as free radical quenchers that eliminate exogenous antioxidants [8].

Traditionally, the lipids used in artificial nutrition are based on ω-6 fatty acid-rich vegetable oils such as soybean oil. These emulsions have raised concerns because of their potential adverse effects involving oxidative stress, inflammation, and immune response, which are probably due to undesirable fatty acids (FA) composition [9]. Recently, fish oil (FO) in lipid emulsions has been introduced as a component of PN. These emulsions contain eicosapentaenoic acid (EPA) and docosahexaenoic acid (DHA) which modulate the synthesis of eicosanoids, the activity of nuclear receptor and nuclear transcription factors, and the production of resolvins, with recognized anti-inflammatory and immunomodulatory effects in critically ill patients [10]. Therefore, they have been associated with less hepatic toxicity and lower levels of low-density lipoprotein triglyceride and C-reactive protein compared to soybean lipid emulsions in post-operative patients [11]. They are likely to reduce infections, the length of the hospital stay, and liver dysfunction without influencing mortality and may be a safe and preferable choice in post-surgery patients [12].

Some authors have shown that these are well tolerated and have a beneficial effect on antioxidant status [13]. In patients with a higher OS, the antioxidant effect of FO-containing emulsions is being investigated specifically because they contain tocopherol and polyunsaturated fatty acids (LC-PUFA) [14].

In the pediatric population there are very few studies of the effects of PN, and most have been conducted in neonates. Recently, in a meta-analysis in preterm neonates, Zhao *et al.* have observed that the level of DHA is efficiently improved by FO lipid emulsions. However, they cannot show any clinical benefit or detrimental effect of using FO in these patients compared to control lipid emulsions [15]. The current study was based on the hypothesis that children with HSCT have alterations in the antioxidant defense system and consequently have an increased vulnerability to oxidative stress. The aims of this study were to evaluate plasma antioxidant levels in children after HSCT using a FO lipid emulsion with potentially beneficial antioxidant effects and to compare the results with those for the classic soybean oil formula used in PN.

## 2. Experimental Section

### 2.1. Subjects

Over 24 months, 17 children with HSCT were consecutively selected and 14 of them completed the study. Two children were excluded because PN was retired before 10 days by a good evolution. Another patient was excluded due to problems with blood extractions. Exclusion criteria were as follows: PN for at least for 10 days, age less than six months or older than 14 years, contraindication to PN, a history of hypersensitivity to egg or soy proteins, severe organ failure, previous liver alterations (transaminase levels twice their normal value and/or total bilirubin >2.5 mg/dL), or a condition requiring the use of a particular lipid emulsion in PN.

Each child was randomly assigned to one of two groups by the pharmacist in the hospital according to a randomization generated by a computer program (SIGESMU^®^). The study was a double-blind trial and other medical interventions were included in the protocol to maintain the uniformity in processes during the study.

Lipid emulsion containers were similar in order to ensure the effectiveness of blinding. The soybean group received the soybean oil formula commonly used in the hospital. The FO group received the formula containing soybean oil, medium-chain triglycerides (MCTs), and ω-3 LC-PUFA.

The study was performed in accordance with the ethical standards of the 1964 Declaration of Helsinki and its later amendments. The work was approved by the Institutional Ethical Committee of the Reina Sofia University Hospital. Written informed consent was obtained from all parents or legal guardians prior to inclusion in the study, and verbal consent was obtained from children when possible. Clinical Trial Registration Number: NCT02199821

### 2.2. Clinical Evaluation

A complete medical history, including the underlying disease (acute leukemia or other hematologic or solid-tumor pathology) and the type of HSCT (autologous or allogeneic) conditioning regimen and engraftment time, was obtained before starting PN.

The scales of Lansky (for <16 years) and Eastern Cooperative Oncology Group (ECOG) were used for clinical and functional assessment of pediatric patients before HSCT. These scales have been designed to evaluate activity (with higher scores in extenuating activities or games, median scores if patients were in bed most of time, or lowest punctuation for minimal activity or exitus). Thus, in the present study, candidates to receive HSCT were those patients with a favorable condition with a Lansky scale >90% or ECOG body between 0–1 [16,17].

Physical examination, including anthropometric measurements such as weight (kg) and height (cm), was performed before and on the completion of PN (day 10 or day 21). Weight and height were measured using standard techniques—a beam balance and a precision stadiometer (Seca)—with participants lightly dressed and barefoot and these values were compared with Spanish reference standards [18]. To evaluate complications of HSCT we recorded daily temperature (fever was defined as a temperature >38 °C); the presence of mucositis, acute graft-*versus*-host disease (GVHD), or veno-occlusive disease (VOD); and renal or neurological alterations. 

### 2.3. Parenteral Nutrition

Each patient was prescribed indexed amounts of energy and the same type and quantities of macronutrients and micronutrients (e.g., vitamins) in relation to their weight and standard recommendations, only by PN, without oral intake. The mean dose for lipids in PN for all the patients was 1.5 g/kg/day up to 2 g/kg/day. The lipid composition of each formula was as follows:

1. *Soybean formula (Intralipid 20%*^®^*)*: 20 g of purified soybean oil per 100 mL. The FA composition was: linoleic acid (18:2ω-6), 52%; alpha-linolenic acid (18:3ω-3), 8%; oleic acid (18:1ω-9), 22%; palmitic acid (16:0), 13%; stearic acid (18:0), 4%; and other FA, 1%. This emulsion contains 240 mg/L of tocopherol (10% is α-tocopherol). The other components were purified egg phospholipids, glycerol, and water for injections.

2. *FO-containing emulsion* *(Lipoplus 20%*^®^*)*: 200 mg/mL (20%) of triglycerides. The composition was 10 g of MCT, 8 g of soybean oil, and 2 g of triglycerides with ω-3 fatty acids per 100 ml. Essential FA: linoleic acid (ω-6), 25.72% (5.14 g/100 mL); alpha-linolenic acid (ω-3), 3.41% (0.68 g/100 mL); oleic acid, 13.44% (2.69 g/100 mL); EPA, 3.69% (0.74 g/100 mL); and DHA, 2.53% (0.51 g/100 mL), ratio ω-3/ω-6 1:2.7. This emulsion contains 190 mg/L of α-tocopherol. The other components were egg lecithin, glycerol, sodium oleate, ascorbylpalmitate, all-rac-alpha-tocopherol, sodium hydroxide, and water for injection.

PN bag except lipid component was protected from ambient light using multilayered bags and a photo resist overwrap from the time of preparation to its administration. After that, opaque infusion systems were used during the infusion. Lipids were repackaged to adjust the prescribed dose for each patient. This manipulation was performed under aseptic conditions in a horizontal laminar flow cabinet according to the protocols of the pharmacy.

The number of days on total PN and on PN combined with enteral nutrition, as well as the time of interruption of PN, *i.e.*, when the patient tolerated appropriate amounts of enteral nutrition, were recorded. Enteral nutrition was started with minimum amounts of liquids with glucose or milk. Omega-3 extra fatty acids were not included in this intake.

### 2.4. Sampling and Biochemical Analysis

Baseline blood samples were collected after a 12-h overnight fast at rest, lying and using a PORT-A-CATH to draw a 3-mL sample in tubes containing EDTA. After centrifugation at 3500× *g* for 10 min, plasma and the buffy coat were removed and pipetted into Eppendorf tubes. The erythrocytes were washed three times with NaCl (0.9%) and lysed with cold water. All samples were processed within 2 h of sampling and divided into aliquots and were then frozen at −80 °C until they were analyzed. All biochemical parameters were routinely evaluated four times (before starting PN, at day 10, at the end of PN, and 10 days after PN). The blood count and general biochemical parameters were assessed in the hospital laboratory using standardized methods and an automatic autoanalyzer (Roche-Hitachi Modular PYD autoanalyzer, Roche Laboratory Systems, Mannheim, Germany).

### 2.5. Antioxidant Biomarker Analysis

Blood antioxidant enzymes and non-enzymatic exogenous biomarkers of the antioxidant defense system were evaluated in both groups at baseline, at 10 days, and at the end of the PN.

### 2.6. Antioxidant Defense Enzymes

Catalase (CAT), glutathione reductase (GR), glutathione peroxidase (GPOX), and superoxide dismutase (SOD) activities in lysed erythrocytes were assayed spectrophotometrically using BIO-TEK Microplate Reader Synergy HT^®^. The hemoglobin concentration in the blood samples was determined spectrophotometrically by the colorimetric cyanmethemoglobin method using Sigma Diagnostic Drabkin reagents. The decomposition of hydrogen peroxide in water at 240 nm was monitored to assess catalase activity [19]. GR activity was determined by spectrophotometric analysis at 340 nm, in which the reduction of oxidized glutathione to reduced glutathione was measured [20]. GPOX activity was measured by assessing the oxidized glutathione produced in the reaction at 340 nm [21]. SOD activity was determined by dichromatic analysis (415/450 nm) using xanthine and xanthine oxidase to produce superoxide radicals [22]. The concentration values of SOD were calculated by extrapolating the absorbance from a curve generated from reference standards. CAT is expressed as nmol/seg·g^−1^ Hb, GPOX and GR as µmol/min·g^−1^ Hb, and SOD is expressed as U/mg Hb. All these enzymatic activities were previously adapted to micromethods.

### 2.7. Non-Enzymatic Antioxidant Exogenous Compounds

The plasma concentrations of retinol (vitamin A), α-tocopherol (vitamin E), β-carotene, coenzyme Q9, and coenzyme Q10 were determined by HPLC coupled to an electrochemical detector (HPLC Waters W2695^®^ EC) after extraction with 1-propanol. β-carotene was also determined after extraction with 1-propanol in an HPLC system attached to a multi-wavelength ultraviolet detector set at 450 nm [23]. All compounds were identified by predetermining the retention times of individual standards.

### 2.8. Statistical Analysis

Sample size was estimated upon primary outcomes as the ratio ω6/ω3 and EPA and alpha-tocopherol, based on previously reported results [14,24,25,26]. Thus, 3–5 children would be needed in each formula group under the assumption of non-inferiority (one-sided test), and accepting an alpha risk of 0.05 and a beta risk of 0.2 in a bilateral contrast test. Dropouts were not included in the calculation.

Data were expressed as the mean or median with standard deviation (SD) or interquartile range (IQR) and 95% confidence intervals (CI). The normal distribution of data was assessed by the Shapiro-Wilk test. Homogeneity of variance was estimated using Levene’s test. Mean values for continuous variables with normal distribution were compared by Student’s *t*-test for unpaired samples and by the Mann-Whitney U test for data with asymmetric distribution. Categorical data were analyzed by χ^2^ or Fisher’s exact test. Comparative analysis between groups was performed according to the lipid formula. Intergroup and intragroup values (group and time) were compared using ANOVA repeated measures with the Sidak correction, except for β-carotene which was corrected with the Mann-Whitney U test and the Wilcoxon test. Correlations between quantitative variables were assessed using the Pearson coefficient for variables with normal distribution and Spearman’s rho analysis for those with asymmetric distribution.

Statistical Package SPSS, version 19.0 by IBM (SPSS Inc, Chicago, Illinois, USA), was used for these analyses.

## 3. Results

Similar demographic characteristics, nutritional status, and complications during the treatment with PN were observed between both groups and are shown in Table 1.

**Table 1:** Demographic and clinical characteristics of 14 children undergoing hematopoietic stem cell transplantation, with parenteral nutrition with two different lipid emulsions based on soybean and fish oil-containing emulsion.

Clinical Parameters	Soybean	FO	*p*
Mean Age (months)	79 ± 56 (28–131)	94 ± 40 (56–131)	0.67
Sex (male/female)	4/3	3/4	1.00
Diagnostic:			0.08
Acute Leukemia	6	2	
Bone marrow aplasia	0	1	
Wiskot Aldrich	0	1	
Solid tumors	1	3	
Type of HSCT			
Autologous	1	3	0.39
Allogenic	6	4	0.56
Complications:			
Mucositis	7	7	0.39
GVHD	4	2	0.43
VOD	1	1	1
Mortality	1	1	1
Total days of PN	16.7 ± 7.36 (10.1–23.8)	16.85 ± 5.52(11.7–22.2)	0.97
Days of PN alone	12.71 ± 8.42 (6.9–20.5)	15.28 ± 6.01(9.7–20.8)	0.52

FO: fish oil-containing emulsion; GVHD: graft-*versus*-host disease; HSCT: hematopoietic stem cell transplantation; PN: parenteral nutrition; VOD: veno-occlusive disease. Data are expressed as mean ± SD (95% confidence intervals).

As previously described, no statistically significant differences were found between the two groups in terms of patient age, sex, primary pathology, or type of HSCT [27]. In addition, patients in both groups showed different degrees of mucositis, GVHD, and VOD. There were also no differences in the total days of PN or in the number of hospitalization days. Eight children (four in each emulsion group) with similar clinical characteristics maintained PN for at least 21 days.

### Antioxidant Status

After 10 days of PN, there were no significant differences between the two groups of children in vitamin A, β-carotene, coenzyme Q9, and coenzyme Q10 plasma concentrations, and in SOD, GR, CAT, or GPOX activities, either before starting PN or at day 10 (Table 2).

**Table 2:** Comparison between blood antioxidant levels in two groups of patients undergoing hematopoietic stem cell transplantation with parenteral nutrition based on soybean- or FO-containing lipid emulsion, at the beginning (baseline) and at day 10 of parenteral nutrition.

Antioxidants	Lipid Emulsions	Baseline	*p*	Day 10	*p*
Vitamin A (mg/L)	Soybean	0.25 ± 0.11	0.25	0.24 ± 0.05	0.89
	FO	0.19 ± 0.06		0.25 ± 0.12	
α-tocopherol (mg/L)	Soybean	5.75 ± 1.04	0.27	6.62 ± 1.34	0.03 *
	FO	6.39 ± 0.94		8.29 ± 1.10	
β-carotene (μg/L)	Soybean ^a^	503.15(2482.12)	0.39	282.86(596.13)	0.75
	FO ^a^	767.94(1344.77)		212.17(206.61)	
Q9 (μg/L)	Soybean	10.99 ± 2.56	0.39	12.55 ± 3.57	0.64
	FO	9.89 ± 1.91		13.96 ± 6.47	
Q10 (μg/L)	Soybean	146.23 ± 92.70	0.51	142.25 ± 64.23	0.64
	FO	120.20 ± 33.82		170.14 ± 141.92	
SOD (U/mg Hb)	Soybean	1.86 ± 0.93	0.69	1.60 ± 0.91	0.34
	FO	2.03 ± 0.56		2.03 ± 0.64	
GR (μmol/min·g^−1^ Hb)	Soybean	2.36 ± 0.80	0.07	2.68 ±0.91	0.49
	FO	3.11 ± 0.52		2.41 ± 0.39	
CAT (nmol/seg·g^−1^ Hb)	Soybean	5.71 ± 1.10	0.67	7.45 ± 1.60	0.09
	FO	5.98 ± 1.17		5.95 ± 1.30	
GPOX (μmol/g Hb)	Soybean	11.94 ± 6.13	0.74	10.13 ± 4.61	0.23
	FO	12.90 ± 4.38		13.26 ± 4.27	
GPOX (μU/g Hb)	Soybean	10.36 ± 5.32	0.74	8.79 ± 4.00	0.23
	FO	11.20 ± 3.80		11.51 ± 3.71	

FO: fish oil-contaning emulsion; Q9: coenzyme Q9; Q10: coenzyme Q10; SOD: superoxide dismutase; GR: glutathione reductase; CAT: catalase; GPOX: glutathione peroxidase; Analysis of variance (ANOVA) with repeated measures for comparisons between groups. Data are expressed as mean ± SD or median (interquartile range); ^a^ U de Mann Whitney. Data are expressed as median (interquartile range); * *p* ≤ 0.05: significant differences between groups of lipid emulsions.

**Figure 1:**
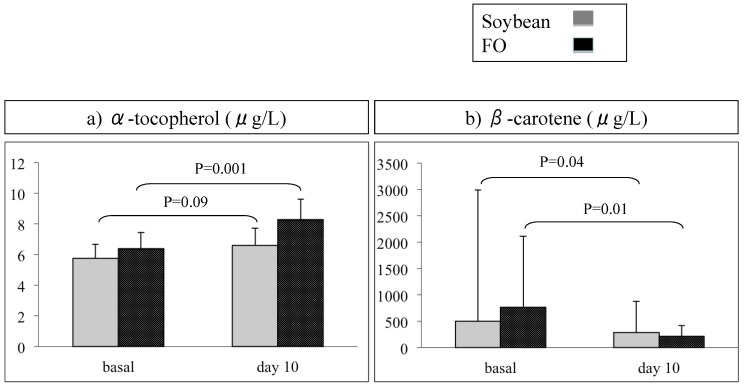
Differences between the levels of α-tocopherol (**a**) and β-carotene (**b**) within 10 days of NP compared to baseline in each group, soybean and fish oil-containing emulsions.

There were no significant differences by time (baseline *vs.* day 10). At day 10 of PN, we observed higher plasma α-tocopherol levels in the group of patients receiving the FO-containing emulsion compared with the group receiving the soybean emulsion. Similarly, when the levels at day 10 were compared with baseline levels, plasma α-tocopherol had increased in the FO group and β-carotene decreased in both groups; this difference was more significant in the group receiving the FO-containing emulsion (Figure 1). Eight patients needed PN for at least 21 days: four in the soybean group and four in the FO group. At 21 days, α-tocopherol plasma levels were higher in the FO group than in the soybean group (*p* = 0.02). No differences in the levels of other antioxidants at 21 days were detected.

## 4. Discussion

In the present study, 14 pediatric patients undergoing HSCT received PN with two different lipid emulsions. They conformed to a homogeneous sample in terms of age, sex, primary disease, type of HSCT, and development of clinical complications. After 10 days of PN, plasma α-tocopherol increased more in the group that received the FO lipid emulsion than in the soybean group.

PN improves the nutritional status of critically ill patients by providing essential substrates and micronutrients [28]. Lipid emulsions provide energy and prevent essential fatty acid deficiency in infants and children requiring PN [29,30]. However, PN has its own complications; for example, excess fat can affect the inflammatory response and increase OS in some patients. PN increases free radical production and decreases liver oxidative metabolism and enzymatic antioxidant defense, primarily in relation to the lipid infusion administered [30,31,32]. An excess of free radical molecules induces lipid peroxidation of PUFAs, which are essential components of cell membranes. This situation leads to impaired function and subsequent cell death [31]. However, in healthy volunteers no significant differences have been found in the immune response or the antioxidant system after an infusion over a short time [33].

Therefore, antioxidant needs seem to be higher in patients receiving PN, and it has been observed that conditioning therapy given to HSCT patients creates high OS and decreases the concentration of antioxidants such as GPOX, α-tocopherol, and β-carotene [2]. In these patients, elevated plasma levels of malondialdehyde (MDA), thiobarbituric acid, and lipid hydroperoxides; decreased levels of vitamin C, α-tocopherol, and β-carotene; and depletion of glutathione (GSH) in the plasma and tissues have been reported. Short-term infusion of PUFA has been shown to increase lipid peroxide formation and decrease plasma glutathione, the most abundant extracellular antioxidant [28]. These can result from low levels of vitamin C and E and increased blood GPOX activity, secondary to the excessive production of free radicals [2,7]. Curiously, Sabuncuoğlu *et al.* [7] reported no changes in the levels of β-carotene and an increase in α-tocopherol after the conditioning regimen. The authors attribute this finding to the administration of vitamin D and other vitamin supplements. Independent of conditioning therapy, Sari *et al.* [6] observed, after an allogeneic peripheral blood progenitor transplant, increased levels of nitric oxide (NO) and decreased antioxidants, including SOD, CAT, and GPOX. High levels of NO in these patients have been linked to the development of acute GVHD [34]. In contrast, our results do not confirm this inhibition of antioxidant enzymes by PN in this type of patient.

Lipid emulsions used in PN are susceptible to lipid peroxidation because of their PUFA content [2,29]. Soybean emulsions rich in ω-6 have traditionally been used in PN. Linoleic acid promotes the generation of eicosanoids derived from arachidonic acid, hence its proinflammatory effect [28,30]. Nonetheless, the clinical benefits of FO-containing emulsion have been described in proinflammatory conditions. Our results did not show differences in the development of clinical complications between the groups. However, plasma levels of linoleic acid [27] and some inflammatory factors increased in the patients who received the soybean emulsion as compared with the FO group.

The effect of the FO-containing emulsion on antioxidant status, especially in patients with OS, is still being debated. It is thought that these emulsions are more susceptible to lipid peroxidation because of their high PUFA content [14]; however, most of the published studies have failed to either confirm this or show otherwise [35]. Several studies have evaluated the impact of soybean and FO-containing emulsion in PN on the antioxidant system following post-operative major abdominal surgery [14,36]. In these studies, after five days of PN, no changes in lipid peroxidation in either group were observed. There was a significant increase in plasma α-tocopherol levels in the group of patients receiving the FO-containing emulsion. Similarly, in our study, β-carotene levels decreased in both groups after 10 days of PN.

Meanwhile, other authors studying the effect of FO-containing emulsion supplemented with vitamin E reported that although α-tocopherol was normalized, in both groups, other unsupplemented antioxidants such as vitamin C, carotene, and selenium were decreased during the administration of PN [8]. Similarly, in our study, β-carotene levels decreased in both groups after 10 days of PN. Furthermore, other authors reported that there have also been changes in the antioxidant defense system after longer periods of PN, with increasing concentrations of α-tocopherol in relation to the administration of FO-containing emulsions after four weeks of treatment [37].

With respect to the increased plasma levels of vitamin E in the FO group, similar results have been published [14,30,36,38]. Vitamin E is an exogenous antioxidant obtained from food or dietary supplements. Its active form, D-α-tocopherol, protects the PUFA of cell membranes from lipid peroxidation [8,39]. In fact, lipid formulas are normally supplemented with vitamin E to limit oxidation. This coadjutant effect could be associated with results of better antioxidant status in patients using new lipid formulas since they contain a higher quantity of α-tocopherol than the soybean formula used in this study. Therefore, possibly, this is a cumulative benefit for patients. In pediatric patients, most investigations have been carried out in neonates and infants. Subjects undergoing total PN have exhibited an increase in oxygen-derived free radicals, primarily in relation to the lipid emulsion used. In a group of infants fed only PN based on soybean oil and supplemented with vitamin C, the levels of vitamin C decreased in term and preterm infants while SOD levels were significantly above baseline only in term infants, probably to counteract the production of MDA, an indicator of free radical activity. Nevertheless, plasma levels of vitamin E increased in both groups receiving total PN and mixed nutrition, parenteral and enteral, in term and preterm infants [31]. In another study using formula supplemented with fish oil and vitamin E, the ratio of ω-3/ω-6 and plasma levels of α-tocopherol increased after eight days and at the end of PN with respect to basal results [38].

Similarly, serum levels of vitamin E have been found to be significantly increased in preterm neonates and children receiving PN based on FO-containing emulsion but did not change in groups fed a soybean lipid emulsion [40,41] or olive oil [42]. In another study, a slight decrease in MDA levels was observed in a group receiving soybean emulsion, but tocopherol levels did not change [38].

## 5. Conclusions

It seems that FO-containing emulsion in long term PN can improve the antioxidant profile by increasing levels of α-tocopherol in children who have a high risk of suffering oxidative stress and metabolic disorders. This study offers new findings about the use of new lipid formulas in PN that can be useful in clinical practice, although randomized pediatric studies with a larger number of patients are needed to identify optimal pharmacological and nutritional treatments or supplements.

## References

[1] Chaudhry M., Ali N. (2015). Reduced-intensity conditioning hematopoietic stem cell transplantation: Looking forward to an international consensus. Blood Res..

[2] Jonas C.R., Puckett A.B., Jones D.P., Griffith D.P., Szeszycki E.E., Bergman G.F., Furr C.E., Tyre C., Carlson J.L., Galloway J.R. (2000). Plasma antioxidant status after high-dose chemotherapy: A randomized trial of parenteral nutrition in bone marrow transplantation patients. Am. J. Clin. Nutr..

[3] Murray S.M., Pindoria S. (2009). Nutrition support for bone marrow transplant patients. Cochrane Database Syst. Rev..

[4] Jain A., Jadhav A.A., Varma M. (2013). Relation of oxidative stress, zinc and alkaline phosphatase in protein energy malnutrition. Arch. Physiol. Biochem..

[5] Owens J.L., Hanson S.J., McArthur J.A., Mikhailov T.A. (2013). The need for evidence based nutritional guidelines for pediatric acute lymphoblastic leukaemia patients: Acute and long-term following treatment. Nutrients.

[6] Sari I., Cetin A., Kaynar L., Saraymen R., Hacioglu S.K., Ozturk A., Kocyigit I., Altuntas F., Eser B. (2008). Disturbance of pro-oxidative/antioxidative balance in allogeneic peripheral blood stem cell transplantation. Ann. Clin. Lab. Sci..

[7] Sabuncuoğlu S., Kuşkonmaz B., UckunÇetinkaya D., Ozgüneş H. (2012). Evaluation of oxidative and antioxidative parameters in pediatric hematopoietic SCT patients. Bone Marrow Transplant..

[8] Mayor-Oxilia R. (2010). Estrés Oxidativo y Sistema de Defensa Antioxidante. Rev. Inst. Med. Trop..

[9] Ren T., Cong L., Wang Y., Tang Y., Tian B., Lin X., Zhang Y., Tang X. (2013). Lipid emulsions in parenteral nutrition: Current applications and future developments. Expert. Opin. Drug. Deliv..

[10] Manzanares W., Langlois P.L., Dhaliwal R., Lemieux M., Heyland D.K. (2015). Intravenous fish oil lipid emulsions in critically ill patients: An updated systematic review and meta-analysis. Crit. Care.

[11] Tian H., Yao X., Zeng R., Sun R., Tian H., Shi C., Li L., Tian J., Yang K. (2013). Safety and efficacy of a new parenteral lipid emulsion (SMOF) for surgical patients: A systematic review and meta-analysis of randomized controlled trials. Nutr. Rev..

[12] Li N.N., Zhou Y., Qin X.P., Chen Y., He D., Feng J.Y., Wu X.T. (2014). Does intravenous fish oil benefit patients post-surgery? A meta-analysis of randomised controlled trials. Clin. Nutr..

[13] Pradelli L., Mayer K., Muscaritoli M., Heller A.R. (2012). *N*-3 fatty acid-enriched parenteral nutrition regimens in elective surgical and ICU patients: A meta-analysis. Crit. Care.

[14] Linseisen J., Hoffmann J., Lienhard S., Jauch K.W., Wolfram G. (2000). Antioxidant status of surgical patients receiving TPN with an omega-3-fatty acid-containing lipid emulsion supplemented with alpha-tocopherol. Clin. Nutr..

[15] Zhao Y., Wu Y., Pei J., Chen Z., Wang Q., Xiang B. (2015). Safety and efficacy of parenteral fish oil-containing lipid emulsions in premature neonates. J. Pediatr. Gastroenterol. Nutr..

[16] Lansky S.B., List M.A., Lansky L.L., Ritter-Sterr C., Miller D.R. (1987). The measurement of performance in childhood cancer patients. Cancer.

[17] Oken M.M., Creech R.H., Tormey D.C., Horton J., Davis T.E., McFadden E.T., Carbone P.P. (1982). Toxicity and response criteria of the Eastern Cooperative Oncology Group. Am. J. Clin. Oncol..

[18] Sobradillo B., Aguirre A., Aresti U., Bilbao A., Fernández-Ramos C., Lizárraga A., Lorenzo H., Madariaga L., Rica I., Ruiz I. (2004). Curvasy Tablas de Crecimiento (Estudios Longitudinaly Transversal).

[19] Aebi H. (1984). Catalase *in vitro*. Methods Enzymol..

[20] Carlberg I., Mannervik B. (1985). Glutathione reductase. Methods Enzymol..

[21] Flohé L., Günzler W.A. (1984). Assays of glutathione peroxidase. Methods Enzymol..

[22] McCord J.M., Fridovich I. (1969). The utility of superoxide dismutase in studying free radical reactions. I. Radicals generated by the interaction of sulfite, dimethylsulfoxide, and oxygen. J. Biol. Chem..

[23] Battino M., Leone L., Bompadre S. (2004). High-performance liquid chromatography-EC assay of mitochondrial coenzyme Q9, coenzyme Q9H2, coenzyme Q10, coenzyme Q10H2, and vitamin E with a simplified on-line solid-phase extraction. Methods Enzymol..

[24] Hartman C., Ben-Artzi E., Berkowitz D., Elhasid R., Lajterer N., Postovski S., Hadad S., Shamir R. (2009). Olive oil-based intravenous lipid emulsion in pediatric patients undergoing bone marrow transplantation: A short-term prospective controlled trial. Clin. Nutr..

[25] D’Ascenzo R., D’Egidio S., Angelini L., Bellagamba M.P., Manna M., Pompilio A., Cogo P.E., Carnielli V.P. (2011). Parenteral nutrition of preterm infants with a lipid emulsion containing 10% fish oil: Effect on plasma lipids and long-chain polyunsaturated fatty acids. J. Pediatr..

[26] Rayyan M., Devlieger H., Jochum F., Allegaert K. (2012). Short-term use of parenteral nutrition with a lipid emulsion containing a mixture of soybean oil, olive oil, medium-chain triglycerides, and fish oil: A randomized double-blind study in preterm infants. J. Parenter. Enter. Nutr..

[27] Baena-Gómez M.A., de la Torre Aguilar M.J., Mesa M.D., Llorente-Cantarero F.J., Pérez Navero J.L., Gil-Campos M. (2013). Effects of parenteral nutrition formulas on plasma lipid profile in children with bone marrow transplantation. Ann. Nutr. Metab..

[28] Siqueira J., Smiley D., Newton C., Le N.A., Gosmanov A.R., Spiegelman R., Peng L., Osteen S.J., Jones D.P., Quyyumi A.A. (2011). Substitution of standard soybean oil with olive oil-based lipid emulsion in parenteral nutrition: Comparison of vascular, metabolic, and inflammatory effects. J. Clin. Endocrinol. Metab..

[29] Krohn K., Koletzko B. (2006). Parenteral lipid emulsions in paediatrics. Curr. Opin. Clin. Nutr. Metab. Care.

[30] Kalish B.T., Le H.D., Gura K.M., Bistrian B.R., Puder M. (2013). A metabolomic analysis of two intravenous lipid emulsions in a murine model. PLoS ONE.

[31] Hasanoğlu A., Dalgiç N., Tümer L., Atalay Y., Cinasal G., Biberoğlu G., Bukan N., Aybar C. (2005). Free oxygen radical-induced lipid peroxidation and antioxidant in infants receiving total parenteral nutrition. Prostaglandins Leukot. Essent. Fat. Acids.

[32] Lespine A., Fernandez Y., Periquet B., Galinier A., Garcia J., Anglade F., Ghisolfi J., Thouvenot J.P. (2001). Total parenteral nutrition decreases liver oxidative metabolism and antioxidant defenses in healthy rats: Comparative effect of dietary olive and soybean oil. J. Parenter. Enter. Nutr..

[33] Versleijen M.W., Roelofs H.M., Rombouts C., Hermans P.W., Noakes P.S., Calder P.C., Wanten G.J. (2012). Short-term infusion of a fish oil-based lipid emulsion modulates fatty acid status, but not immune function or (anti)oxidant balance: A randomized cross-over study. Eur. J. Clin. Invest..

[34] Choi I.C., Fung P.C., Leung A.Y., Lie A.K., Liang R. (2001). Plasma nitric oxide is associated with the occurrence of moderate to severe acute graft-versus-host disease in haemopoietic stem cell transplant recipients. Haematologica.

[35] Kelley N.S., Yoshida Y., Erickson K.L. (2014). Do *n*-3 polyunsaturated fatty acids increase or decrease lipid peroxidation in humans?. Metab. Syndr. Relat. Disord..

[36] Wichmann M.W., Thul P., Czarnetzki H.D., Morlion B.J., Kemen M., Jauch K.W. (2007). Evaluation of clinical safety and beneficial effects of a fish oil containing lipid emulsion (Lipoplus, MLF541): Data from a prospective, randomized, multicenter trial. Crit. Care Med..

[37] Klek S., Chambrier C., Singer P., Rubin M., Bowling T., Staun M., Joly F., Rasmussen H., Strauss B.J., Wanten G. (2013). Four-week parenteral nutrition using a third generation lipid emulsion (SMOF lipid)—A double-blind, randomised, multicentre study in adults. Clin. Nutr..

[38] Tomsits E., Pataki M., Tölgyesi A., Fekete G., Rischak K., Szollár L. (2010). Safety and efficacy of a lipid emulsion containing a mixture of soybean oil, medium-chain triglycerides, olive oil, and fish oil: A randomised, double-blind clinical trial in premature infants requiring parenteral nutrition. J. Pediatr. Gastroenterol. Nutr..

[39] Pham-Huy L.A., He H., Pham-Huy C. (2008). Free radicals, antioxidants in disease and health. Int. J. Biomed. Sci..

[40] Skouroliakou M., Konstantinou D., Koutri K., Kakavelaki C., Stathopoulou M., Antoniadi M., Xemelidis N., Kona V., Markantonis S. (2010). A double-blind, randomized clinical trial of the effect of omega-3 fatty acids on the oxidative stress of preterm neonates fed through parenteral nutrition. Eur. J. Clin. Nutr..

[41] Goulet O., Antébi H., Wolf C., Talbotec C., Alcindor L.G., Corriol O., Lamor M., Colomb-Jung V. (2010). A new intravenous fat emulsion containing soybean oil, medium-chain triglycerides, olive oil, and fish oil: A single-center, double-blind randomized study on efficacy and safety in pediatric patients receiving home parenteral nutrition. J. Parenter. Enter. Nut..

[42] Deshpande G., Simmer K., Deshmukh M., Mori T.A., Croft K.D., Kristensen J. (2014). Fish Oil (SMOFlipid) and Olive Oil Lipid (Clinoleic) in Very Preterm Neonates. J. Pediatr. Gastroenterol. Nutr..

